# Allicin alleviates lead-induced hematopoietic stem cell aging by up-regulating PKM2

**DOI:** 10.1042/BSR20190243

**Published:** 2019-07-05

**Authors:** Shi-zhong Cai, Li-na Zhao, Jun Liu, Yi-ting Ji, Xiao-yan Shi, Zhou-rui Ma, Xiao-hua Lv, Ke Chen, Yan Chen

**Affiliations:** 1 Department of Child and Adolescent Healthcare, The Children’s Hospital of Soochow University, Suzhou 215021, Jiangsu, China; 2 Center for Clinical Laboratory, The First Affiliated Hospital of Soochow University, Suzhou 215006, Jiangsu, China; 3 Jiangsu Institute of Hematology, The First Affiliated Hospital and Collaborative Innovation Center of Hematology, Soochow University, Key Laboratory of Thrombosis and Hemostasis, Ministry of Health, Suzhou 215006, Jiangsu, China; 4 Department of Cineplastic Surgery, The Children’s Hospital of Soochow University, Suzhou 215021, Jiangsu, China; 5 Department of Pharmacology, Guangdong Medical University, Zhanjiang 524023, Guangdong, China; 6 Department of Spine Surgery, Shenzhen People’s Hospital, Jinan University Second College of Medicine, Shenzhen 518020, Guangdong, China

**Keywords:** Aging, Allicin, Hematopoietic stem cell, Lead, PKM2

## Abstract

Hematopoietic stem cells (HSCs) aging is associated with hematopoietic dysfunction and diseases. Our previous study showed that lead exposure induced a functional decline in HSCs. Allicin, a chemical extracted from the garlic (*Allium sativum L.*), has been reported to have antioxidative and anti-inflammatory effects. However, the biological activities of allicin on lead-induced toxicity, especially in the hematopoietic system, remain unclear. Here, we found that lead exposure elicited aging phenotypes in HSCs, including perturbed cell quiescence, disabled self-renewal function and colony-forming ability, and myeloid-biased differentiation, all of which contributed to significant hematopoietic disorders in mice. Intragastric administration of allicin substantially ameliorated lead-induced HSCs aging phenotypes *in vivo*. Lead exposure induced a peroxide condition in HSCs leading to DNA damage, which reduced expression of the glycolytic enzyme pyruvate kinase M2 isoform (PKM2), a phenotype which was significantly ameliorated by allicin treatment. These findings suggested that allicin alleviated lead-induced HSCs aging by up-regulating PKM2 expression; thus, it could be a natural herb for preventing lead toxicity.

## Introduction

Aging is a physiological process that is driven by both intrinsic and extrinsic factors. Hematopoietic stem cells (HSCs), the common progenitor of all blood cells, comprise one of the best characterized stem cell populations in the body and the only stem cell that is clinically applied for disease treatment, such as breast cancer, leukemia, and congenital immunodeficiencies [1]. It has been reported that the capacity to generate lymphoid cells declines as HSCs age, and impaired hematopoietic systems are observed in aged mice [2], which also show enhanced myelopoiesis and suppressed lymphoid lineages [3]. Importantly, HSCs aging is also implicated in chronic inflammation, and declined immune function, which leads to the onset of serious diseases [4]. However, there are no potential therapies for delaying HSCs aging.

Lead is a silvery white metal that widely exists in living surroundings. It has been reported that lead can be found in gasoline, lead-based paints, and cans that contain food or beverages. Exposure to high lead levels can induce significant public health problems, such as neurotoxicity, reproductive toxicity, liver toxicity, kidney damage, immunotoxicity, and hematological dysfunction [5]. The population most susceptible to these effects is preschool age children, due to the ease of exposure in their blood and brain [6,7]. Previous studies have identified that the hematological system is an important target of lead-induced toxicity, and that lead exposure impairs HSCs function by inducing HSCs aging or apoptosis [8]. Dysfunctional HSCs result from an imbalance in erythrocytes, while due to the fact that erythrocytes exist in the various organs of the body, their status will also affect other systems [9].

Garlic (*Allium sativum*) is widely consumed, and mounting studies have identified that garlic showed ameliorating role on diseases, such as cardiovascular disease and cancer [10,11]. A research reported that those protective effects were associated with allicin [12], which is the product of interactions between alliinase and alliin and is emitted by cutting and crushing garlic cloves. Recently, allicin has been widely studied for its value of potential medical properties. For example, allicin is considered a potential therapeutic agent for osteoarthritis [13]. Additionally, the antioxidant, immunomodulatory, anti-inflammatory, antidiabetic, and antigenotoxic effects of allicin have also been reported [14–17]. Allicin performed ameliorative effects on *Pasteurella multocida* infected rabbits [15]. The nephroprotective effects of allicin was observed on cisplatin-induced toxicity [17]. The beneficial role of allicin was employed against doxorubicin-induced cardiotoxicity [18]. In addition, allicin also showed antioxidant role on *Nile tilapia* [19] and stem cells [20]. Moreover, allicin also showed anti-aging effects on H_2_O_2_-induced human umbilical vein endothelial cells (HUVECs) [21]. However, whether allicin ameliorates lead-induced HSCs aging, and the potential underlying mechanism are still unclear.

In the present study, we first examined the phenotypes associated with aging HSCs that correlated with lead treatment. Then, allicin was applied to decipher its potential role in lead-induced aging. Our findings provide significant data for the clinical prevention and treatment of lead-induced HSCs aging.

## Materials and methods

### Drugs, animals, and ethics statement

Allicin (purity > 98%, Catalog: MB5783) were purchased from Dalian Meilun Biotech, Co., Ltd (Dalian, China). C57BL/6 mice were purchased from Shanghai Laboratory Animal Center, Chinese Academy of Science (Shanghai, China). All mice were male and weighted 20 ± 2 g. For 7 days before treatment, mice were housed in ventilated cages and given free access to standard feed and distilled drinking water under a 12-h light/dark cycle. For treatment, the mice were divided into four groups (*n*=6 per group) as is shown in Figure 3A. In the lead-exposed group, mice were fed 0.2% (wt/v) lead acetate (Sigma–Aldrich, St. Louis, MO) in water for 8 weeks, while control mice were maintained in the same environment but without the addition of lead acetate to the water. In the lead/allicin group, mice were fed the same volume of drinking water containing 0.2% (wt/v) lead acetate and a final dose of 30 μg/kg/day allicin dissolved in 200 μl ethyl alcohol (Meilunbio, Dalian, China) for 8 weeks, while allicin mice were maintained in the same environment but without lead acetate water. On the last day of the 8 weeks, all mice were killed by cervical dislocation for subsequent analyses. All experiments were approved by the Animal Care and Use Committee of our institute, and all animal procedures were conducted in accordance with existing regulations.

### Flow cytometry and cell sorting

For LSK (Lin^−^sca-1^+^c-kit^+^) cells soring, lineage cells are plotted as the population of B220^+^ (eBioscience), CD3^+^ (eBioscience), and Myeloid (Gr-1^+^Mac-1^+^) (eBioscience) cells in bone marrow mononuclear cells by FACS analysis using a BD FACS Aria I (BD Bioscience). After lineage depletion, lineage-negative cells were stained with anti-Sca-1 (eBioscience) and anti-c-Kit (eBioscience) and subjected to FACS analysis to sort the population of LSK cells (gated as Lin^−^/Sca-1^+^/c-Kit^+^) using a BD FACS Aria I (BD Bioscience). Lymphocytes from bone marrow were plotted as the population of CD4^+^CD8^+^ (BD Bioscience).

### Colony-forming assay

For long-term cultures, HSCs were cultured in six-well plates at a density of 1 × 10^3^ cells/well. After 2 weeks in culture, cells were harvested and used for hematopoietic colony-forming assays. Colony-forming assays were performed with 20 ng/ml SCF (PeproTech EC Ltd., Rocky Hill, NJ, U.S.A.), 20 ng/ml IL-3 (PeproTech EC Ltd.), 2 U/ml Epo (Chugai Pharmaceutical Co., Ltd.), and 20 ng/ml G-CSF (Kirin Brewery Co., Ltd.). Colonies were fixed after 12 days, and captured by microscopy (Leica, Germany) according to previous study [22]. The densities were analyzed by ImageJ software.

### Serial competitive bone marrow transplantation

Four to six weeks old C57BL/6 background mice were used for serial competitive bone marrow transplantation (BMT); 1 × 10^6^ bone marrow cells isolated from 4–6 weeks old Ly5.2^+^ mice were mixed with 1 × 10^6^ bone marrow cells isolated from lead-exposed Ly5.1^+^ mice and injected into the retro-orbital sinus of irradiated recipient Ly5.1^+^ mice in 200 μl in PBS. Primary transplanted mice were killed after 20 weeks and 1 × 10^6^ BM cells from each recipient mouse were injected into a secondary recipient Ly5.1^+^ mouse. Serial BMT experiments were repeated three times.

### Intracellular ROS measurements

Intracellular ROS was detected using 2′,7′-dichlorofluorescein diacetate (DCFH-DA) as described previously [23]. Briefly, 5 × 10^5^ cells were washed with PBS and incubated with 300 nM DCFH-DA (Sigma-Aldrich, St. Louis, MO, U.S.A.) at 37°C for 30 min. After incubation, cells were washed, resuspended in PBS, and then cultured with 50 mM H_2_O_2_ for 30 min. Fluorescence intensities were measured with a spectrophotometer at an excitation wavelength of 485 nm and an emission wavelength of 525 nm.

### Western blotting

For Western blots, proteins were isolated from cells or tissues using RIPA buffer (Beyotime, Shanghai, China). Proteins were quantitated using the BCA protein assay kit (Beyotime, Shanghai, China). SDS/PAGE was performed to separate proteins, and equal amounts of proteins were transferred to PVDF membranes, which were incubated with the primary antibodies anti-γ-H2XA, anti-pyruvate kinase M2 isoform (PKM2) (1:1000), and anti-GAPDH (1:500; Abcam, Cambridge, U.K.) at 4°C. After this step, membranes were washed with TBST, incubated with goat anti-rabbit IgG secondary antibody, (1:1000; Beyotime) at room temperature for 1 h, and then immunoreactive bands were analyzed using ImageJ software.

### Real-time PCR

Total RNA was isolated from cells or tissues using a Total RNA Extraction Kit (Promega, Madison, WI, U.S.A.). RNA quality was determined using 0.8% agarose gel electrophoresis. A total of 2 μg of RNA was used for reverse transcription, and the synthesized cDNA was used for real-time PCR. Real-time PCR was performed using SYBR Premix E Taq (Takara Bio) in a Real-Time PCR System Mx3000P (Stratagene) following the manufacturer’s instructions. Relative mRNA expression was calculated by the 2^−ΔΔ*C*^_t_ method. GAPDH was used to normalize PKM2 expression. The primers used in the present study were as follows: PKM2-F: 5′-AGAATTCCATGGGACACGTGTGTG-3′; PKM2-R: 5′-TTCTCGAGTCCGCCAAAGATTG-3′; GAPDH-F: 5′-CATGTTTGTGATGGGCGTGAA-3′; and GAPDH-R: 5′-GATGACTTTGGCTAGAGGAGC-3′.

### Enzyme-linked immunosorbent assay

Peripheral blood was collected, and levels of IL-6 and TNF-α were analyzed by enzyme-linked immunosorbent assay (ELISA) according to the manufacturer’s protocol (eBioscience, San Diego, CA, U.S.A.). Optical densities were quantitated at 450 nm using a microplate reader. Concentrations were calculated according to the standard curve.

### Statistical analysis

Data are presented as mean ± SD. All data were processed using SPSS v19.0 software (IBM, Armonk, NY, U.S.A.). Student’s *t* test or one-way ANOVA was used to analyze the significance of differences as appropriate. A *P*-value <0.05 was considered significant.

## Results

### Lead-exposed mice exhibited a perturbed hematopoiesis and aging phenotype

**Figure 1:**
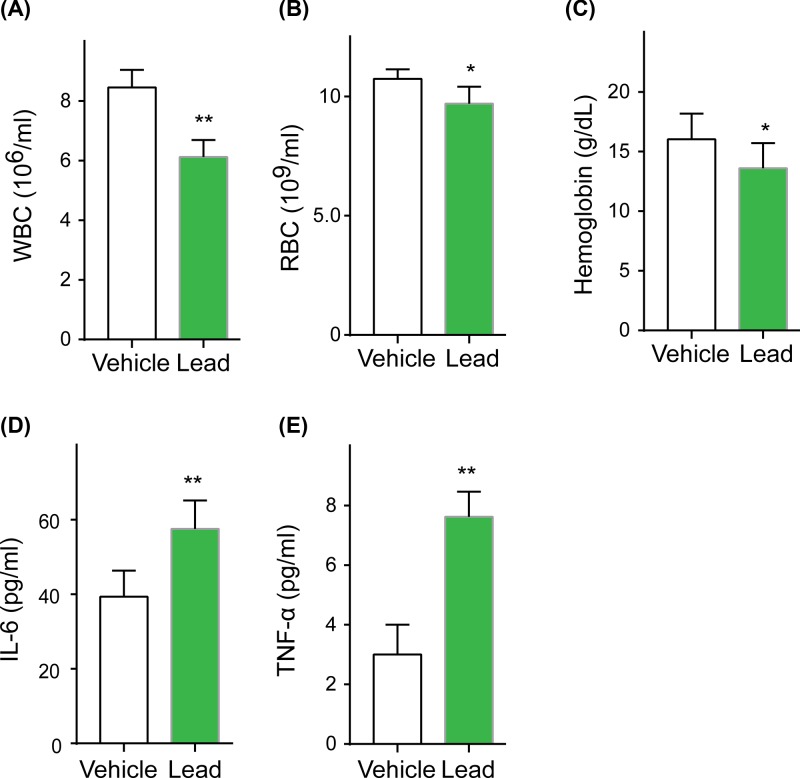
Lead-exposed mice exhibited a perturbed hematopoiesis and aging phenotype C57BL/6 mice were exposed with lead acetate for 8 weeks. Quantification of the WBC level (**A**), the RBC level (**B**), the hemoglobin level (**C**), the inflammatory factors IL-6 (**D**) and TNF-α (**E**) in lead exposed mice. All data are shown as mean (M) ± SD, *n*=6, *P<0.05, **P<0.01.

To determine whether lead exposure affected hematopoiesis, we monitored the hematological parameters of lead-exposed mice. Consistent with previous studies [8], we found that lead exposure led to a significant reduction in white blood cells (WBCs) (Figure 1A), red blood cells (RBCs) (Figure 1B) and hemoglobin (Figure 1C). Organismal aging is associated with chronic low-grade inflammatory senescence-associated secretory phenotype (SASP), and inflammatory factors IL-6 and TNF-α are the biomarkers of SASP [24,25]. In this study, the inflammatory factors IL-6 (Figure 1D) and TNF-α (Figure 1E) were significantly increased in lead-exposed mice, indicating that lead exposure promoted SASP development.

### HSCs from lead-exposed mice exhibited premature aging

**Figure 2:**
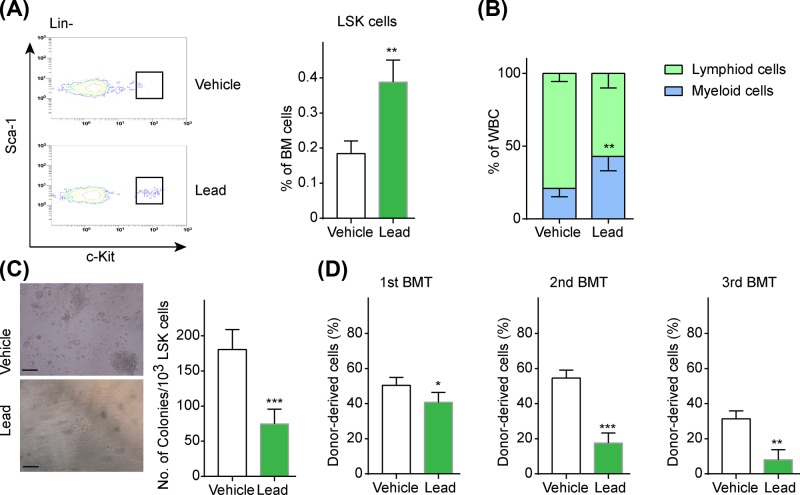
HSCs from lead-exposed mice exhibited premature aging (**A**) LSK cells population in vehicle and lead exposed mice, (**B**) the cell ratio of lymphoid cells and myeloid cells in vehicle and lead exposed mice. (**C**) Colony forming ability, (**D**) self-renewal function of HSCs that isolated from vehicle and lead-exposed mice by serial competitive transplantation. All data are shown as mean (M) ± SD, *n*=6, *P<0.05, **P<0.01, ***P<0.001, scale bar = 50 μm.

We previously found that low-level lead exposure elicited HSCs aging, while, relatively high levels of lead exposure induced HSCs apoptosis [8]. Aged HSCs had perturbed stem cell quiescence which led to diminished cell cycle progression and myeloid-biased differentiation in bone marrow cellularity. Next, we determined whether the distorted hematological parameters were contributed by an imbalance in quiescent HSCs. Lead-exposed mice showed perturbed cell quiescence of HSCs, as indicated by the increased cell proliferation of LSK populations (Figure 2A). Additionally, we found that lead-exposed mice exhibited an increase in the frequency of myeloid cells and reduction in lymphoid cells (Figure 2B). Aged HSCs have functional defects including decreased self-renewal and colony-forming ability. Thus, we determine whether lead exposure impacted self-renewal of HSCs. We found that LSK cells derived from lead-exposed mice had significantly reduced colony-forming ability (Figure 2C). Serial BMT is a well-accepted method to evaluate HSCs function. Serial transplantation results showed that lead-exposed LSK cells had decreased self-renewal ability (Figure 2D). These results indicated that lead exposure promoted a premature aging phenotype and caused a defect in HSCs self-renewal.

### Allicin alleviated the aging features induced by lead exposure

**Figure 3:**
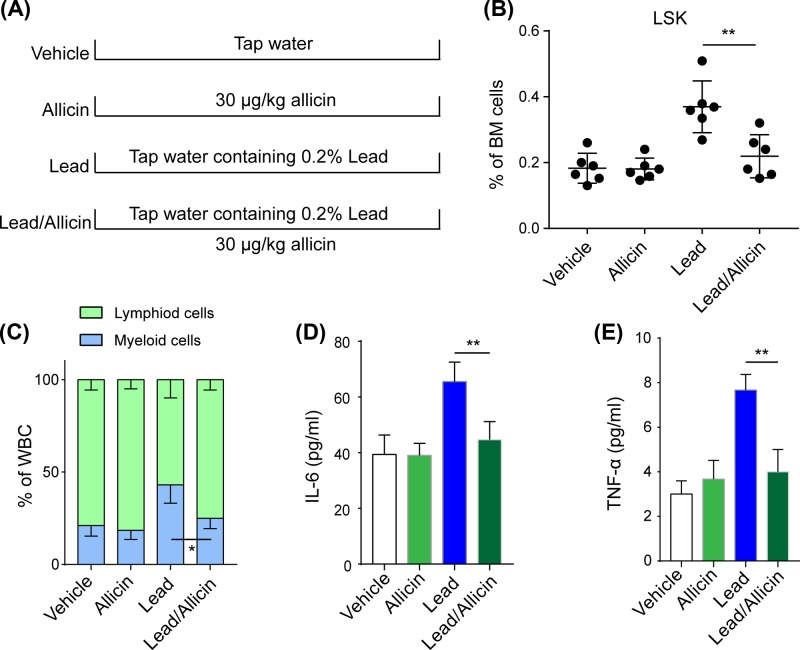
Allicin alleviated the aging features induced by lead exposure (**A**) Schematic chart of mouse exposure to lead and allicin in different groups. (**B**) the ratio of LSK cells in BM cells from different groups; (**C**) the ration of lymphoid cells to myeloid cells in whole BM cells from different groups; (**D**-**E**) the inflammatory factors IL-6 (**D**) and TNF-α (**E**) in peripheral blood in different groups. All data are shown as mean (M) ± SD,*n*=6, *P<0.05, **P<0.01.

It has been reported that allicin has antioxidative and anti-inflammatory effects in multiple tissues and cellular processes. Next, we evaluated the effects of allicin on lead-induced aging phenotypes. Mice were separately fed 0.2% (wt/v) lead or 30 μg/kg allicin by intra-gastric administration. After 8 weeks, HSCs were isolated from mice of the Vehicle, Allicin, lead, and lead/Allicin groups, respectively (Figure 3A). The results showed that allicin treatment ameliorated lead-induced increases in HSC numbers (Figure 3B). Additionally, allicin significantly reversed the lead-induced imbalance in the differential population of myeloid and lymphoid cells in the bone marrow (Figure 3C). Allicin treatment significantly ameliorated SASP features by reducing IL-6 (Figure 3D) and TNF-α (Figure 3E) levels in peripheral blood. These results indicated that allicin alleviated lead-induced aging phenotypes.

### Allicin attenuated the defective lead-induced self-renewal function of HSCs

**Figure 4:**
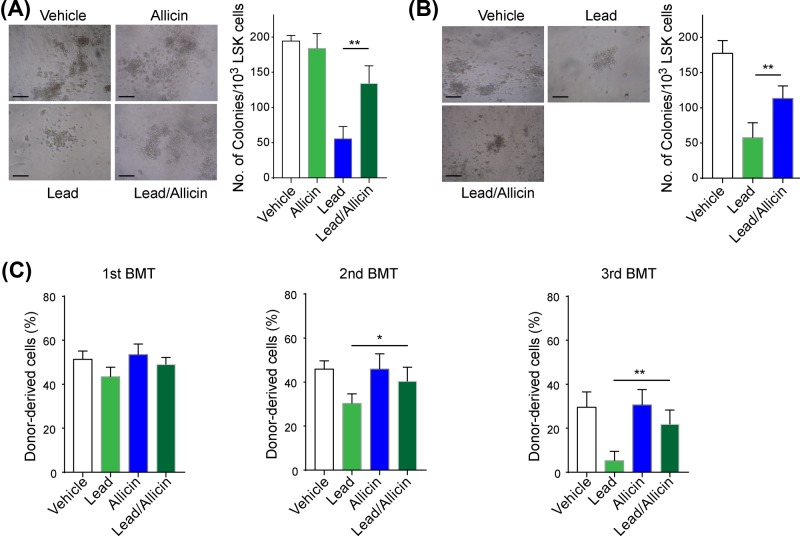
Allicin attenuated lead-induced self-renewal function of HSCs LSK cells isolated from mice (*n*=6) in different groups (vehicle, allicin, lead, lead/allicin) were collected: (**A**) colony forming ability of LSK cells; LSK cells were isolated from C57BL/6 mice and followed by lead or lead/allicin exposure for 72 h. (**B**) colony forming ability of LSK cells, (**C**) self-renewal function of HSCs that isolated from different group mice (*n*=6) performed by serial competitive transplantation. All data are shown as mean (M)±SD, *p<0.05, **P<0.01, scale bar=50 μm. The experiments were performed in triplicate.

Next, *in vivo* and *in vitro* experiments were performed to examine the effect of allicin on HSCs functions. As expected, the colony-forming ability of LSK cells was significantly improved in allicin-treated lead-exposed mice compared with lead-exposed mice (Figure 4A). In the HSCs model *in vitro*, allicin treatment dramatically alleviated the lead-induced decrease in LSK colony forming (Figure 4B). Next, BM competitive serial transplant was performed to confirm the results. LSK cells were isolated from different group of mice and transplanted into the recipient mice (pre-exposed to lethal dose irradiation). After three repeated transplantations, we found that allicin-treated lead-exposed LSK cells had increased self-renewal ability compared with lead-exposed LSK cells (Figure 4C). These data revealed that allicin ameliorated the defective lead-induced self-renewal function of HSCs.

### Allicin attenuated lead-induced increased cellular ROS and DNA damage

**Figure 5:**
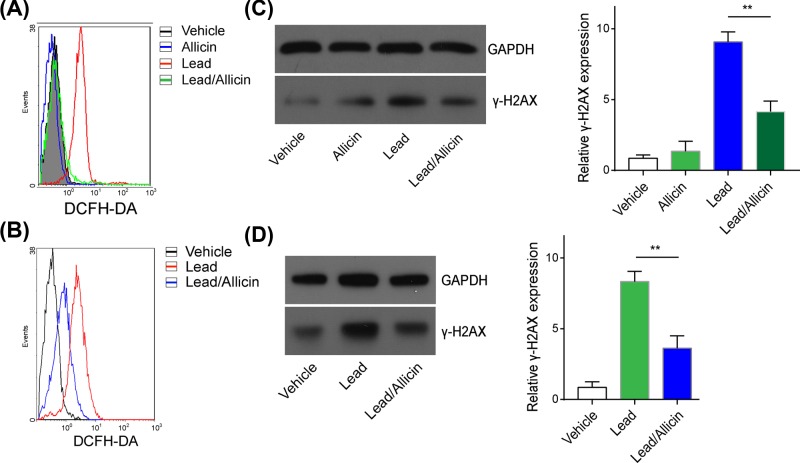
Allicin attenuated lead-induced increased cellular ROS and DNA damage (**A**) intracellular ROS levels were determined in LSK cells that isolated from mice (*n*=6) in different groups. (**B**) intracellular ROS levels were determined in LSK cells pretreated by lead or lead/allicin. (**C**) DNA damage level (γ-H2XA) were determined in LSK cells isolated from mice (*n*=6) in different groups. (**D**) DNA damage level (γ-H2XA) were determined in LSK cells pretreated with lead or lead/allicin. All data are shown as mean (M)±SD, **P<0.01. The experiments were performed in triplicate.

We previously found that lead exposure perturbs the hematopoietic balance of HSCs by inducing cellular mitochondria defects and increased intracellular ROS generation [8]. ROS is important in various biological processes, and excessive ROS affects intracellular redox states and further promotes aging [26]. ROS accumulation has been previously identified to contribute to HSCs aging [27]. In this study, we also found that lead treatment enhanced ROS generation in LSK cells of lead-exposed mice, which was notably reduced by allicin treatment (Figure 5A). In the HSCs model *in vitro*, allicin treatment dramatically alleviated the lead-induced increase in ROS generation (Figure 5B). Previous study showed that Plumbum could generate DNA damage by elevating ROS level in human cells [28]. Next, we investigated whether DNA damage contributes to lead-induced HSCs aging. In the presence of higher level of ROS in lead-induced HSCs, we observed the DNA damage in HSCs and found that the expressions of γ-H2AX, an indicator of DNA damage, were significantly elevated in lead-induced HSCs compared with the control *in vitro* and *in vivo* (Figure 5C,D). More importantly, allcin treatment also effectively alleviates DNA damage in lead-induced aged HSCs (Figure 5C,D). These data indicated that lead-induced HSCs aging was attributed to the ROS-elicited DNA damage, and allicin attenuated lead-induced increased cellular ROS and DNA damage of HSCs.

### Allicin alleviated lead-induced HSCs aging by up-regulating PKM2

**Figure 6:**
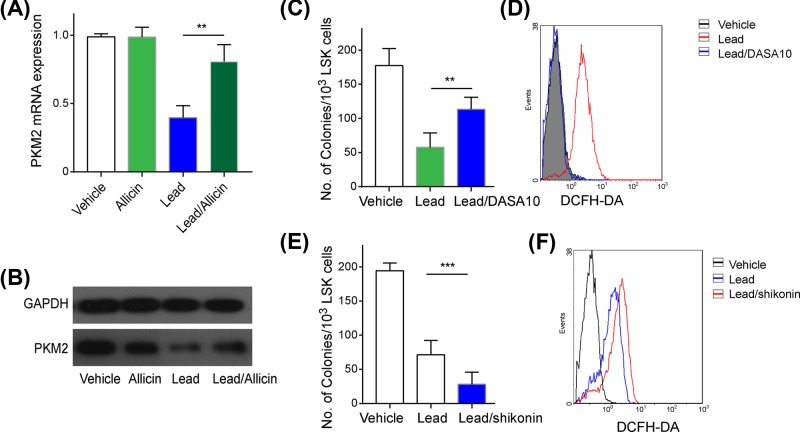
Allicin alleviated lead-induced HSCs aging by upregulating PKM2 (**A,B**) mRNA and protein expression of PKM2 on LSK cells that isolated from mice (*n*=6) in different groups were determined; (**C**) colony forming ability of LSK after stimulation by the PKM 2 activator DASA10; (**D**) cellular ROS accumulation in LSK after stimulation by the PKM2 activator DASA10; (**E**) colony forming ability of LSK treated by PKM2 inhibitor shikonin; (**F**) cellular ROS accumulation in LSK treated by PKM2 inhibitor shikonin. All data are shown as mean (M)±SD, **p<0.01, ***p<0.001. The experiments were performed in triplicate.

To explore the potential mechanism through which allicin alleviated lead-induced aging, we investigated the glycolytic enzyme PKM2, a kinase involved in mediating intracellular ROS levels [18]. We first measured PKM2 expression in mouse LSK cells. The results revealed that lead exposure significantly decreased PKM2 expression, while allicin treatment promoted its expression at the mRNA (Figure 6A) and protein levels (Figure 6B). To determine whether PKM2 specifically mediated the mechanism through which allicin alleviated lead-induced aging, HSCs were pretreated with the PKM2 activator DASA-10 followed by lead exposure. These results revealed that when HSCs were pretreated with DASA-10, there was increased colony formation (Figure 6C) and reduced ROS in LSK cells compared with cells exposed by lead alone (Figure 6D). Consistently, inhibition of PKM2 in HSCs by the PKM2 inhibitor shikonin significantly decreased colony-forming ability (Figure 6E) and promoted extra ROS generation (Figure 6F). These results demonstrated that allicin alleviated lead-induced HSCs aging by up-regulating PKM2.

## Discussion

HSCs’ functions have been widely studied by many groups, and properties of these cells, such as their multi-potency, self-renewal, and quiescence, are required for HSCs to maintain their proper function in the body. HSCs aging is thought to be associated with declined HSCs function (impaired self-renewal and regeneration) and decreased immune responses. Markers associated with HSCs function, such as WBC, RBC, and hemoglobin levels are dramatically altered [29] in aging HSCs. In the present study, lead exposure significantly decreased WBC, RBC, and hemoglobin levels, indicating that lead exposure weakened the function of HSCs. Aging is associated with increased inflammation [30]. Our study showed that lead exposure induced a significant increase in inflammatory factors, such as IL-6 and TNF-α, indicating that lead exposure induced HSCs aging. Additionally, the high levels of LSK, increased number of myeloid cells and decreased number of lymphoid cells after lead exposure demonstrated that lead exposure promoted HSCs activation. Taken together, these data show that lead exposure promoted HSCs aging *in vivo*. Furthermore, HSCs isolated from mice that were given lead showed significantly decreased colony-forming capacity, indicating that lead exposure decreased HSCs’ functions *in vitro*.

Recently, studies showed that some food-derived small molecular and extracts that from vegetable and plants could extend lifespan and delay aging in the model organisms [31–33]. A broad research strategy has been applied to explore therapeutic interventions that might revert or at least ameliorate HSCs aging. For example, pharmacologically inhibiting Cdc42 could rejuvenate aged HSCs [2]. Additionally, antioxidants and rapamycin have also been identified to ameliorate HSCs aging [34,35]. It is widely accepted that protecting HSCs from ambient stress contributes to maintaining the bone marrow niche [36]; thus, mechanisms for maintaining HSCs functions have been widely reported. For example, a previous study identified that the self-renewal capacity of HSCs was related to ATM-mediated inhibition of oxidative stress [37]. The p38MAPK pathway activation that is induced by ROS enhances HSCs exhaustion; therefore, antioxidant treatment could prolong the lifespan of HSCs [35]. In this study, both *in vitro* and *in vivo* experiments showed that allicin alleviated lead-induced HSCs aging, indicating that allicin might serve as an important herb for the clinical prevention of HSCs aging. However, the precise clinical mechanisms need further exploration.

PKM2 is the pyruvate kinase isoform that was first discovered as a cytosolic glycolytic enzyme. Recently, PKM2 has been reported to conduct various biological functions in extracellular secretion, mitochondrial location, and nuclear translocation [38]. Further, PKM2 is expressed in embryonic tissues and tumor cells and is further responsible for catalyzing glycolysis during tissue regeneration and tumor formation [39–41]. For example, PKM2 is massively expressed in human cancers [42–44]. Additionally, PKM2 expression is correlated with ROS production in yolk sac HSCs [45]. In this study, lead exposure decreased both PKM2 mRNA and protein levels, while allicin treatment reversed this effect of lead. Additionally, the increased ROS accumulation that was induced by lead exposure was significantly reduced by the PKM2 activator, DASA10. Moreover, LSK colony-forming ability was also altered following DASA10 treatment, and decreasing PKM2 levels promoted ROS accumulation and further accelerated HSCs aging, indicating that PKM2 plays a vital role in the allicin-mediated alleviation of lead-induced HSCs aging.

In conclusion, the present study revealed the potential ameliorating effect of allicin against lead-induced HSCs aging. The primary results are that lead exposure decreased the self-renewal capacity of HSCs and enhanced HSCs aging, while allicin alleviated lead-induced HSCs aging by up-regulating PKM2. The present study was the first to uncover the protective mechanism of allicin on lead-exposed HSCs, which might lead to the development of a therapy for aged HSCs and lead toxicity.

## Abbreviations

BMT: bone marrow transplantation
DCFH-DA: 2′,7′-dichlorofluorescein diacetate
G-CSF: Granulocyte colony-stimulating fctor
HSC: hematopoietic stem cell
LSK: Lin^−^sca-1^+^c-kit^+^
PKM2: pyruvate kinase M2 isoform
RBC: red blood cell
ROS: Reactive oxygen species
SASP: senescence-associated secretory phenotype
TBST: Tris buffered saline tween
WBC: white blood cell
